# Association between vitamin D receptor gene polymorphism (rs731236) and aggrecan gene VNTR polymorphism with the risk of lumbar intervertebral disc degeneration

**DOI:** 10.22088/cjim.13.2.418

**Published:** 2022

**Authors:** Kaveh Haddadi, Mohammad Sahebi, Abdolkarim Mahrooz, Masoud ShayestehAzar, Mohammad Bagher Hashemi-Soteh

**Affiliations:** 1 Department of Neurosurgery, Orthopedic Research Center, Mazandaran University of Medical Sciences, Sari, Iran; 2 Student Research Committee, Mazandaran University of Medical Sciences, Sari, Iran; 3 Molecular and Cell Biology Research Center, Immunogenetics Research Center, Faculty of Medicine, Mazandaran University of Medical Sciences, Sari, Iran; 4 Orthopedic Research Center, Mazandaran University of Medical Sciences, Sari, Iran.; 5 Immunogenetics Research Center, Molecular and Cell Biology Research Center, Faculty of Medicine, Mazandaran University of Medical Sciences, Sari, Iran

**Keywords:** Intervertebral disk degeneration, Polymorphism, VNTR of aggrecan gene, VDR gene, VAS

## Abstract

**Background:**

Low back pain is one of the most common causes of referral to physicians. Lumbar disc degeneration (LDD) is the main cause of back pain in different countries. It seems that genetic factors are more effective than environmental factors in the developing of degenerative phenomena. The aim of this investigation, therefore, was to study the association of the aggrecan gene (*ACAN*) variable number tandem repeat (VNTR) and the vitamin D receptor (VDR) rs731236 (*TaqI*) polymorphisms, with lumbar intervertebral disc degeneration in a population in the North of Iran.

**Methods:**

In this study, 55 patients with symptomatic intervertebral disk degeneration and 55 control subjects were included. VDR gene polymorphism was genotyped by PCR-based RFLP. The isolated DNA was used to genotype the VNTR of ACAN gene via conventional PCR.

**Results:**

For VDR gene polymorphism, the CC genotype (OR=5.337, P=0.019) was significantly higher among the patients compared with the controls, revealing a higher frequency of the C allele in patients compared with controls (OR=2.707, P=0.005). The lower number of frequent repetitions in the VNTR aggrecan gene was associated with a six-time increase of lumbar disc degeneration. Also, high BMI can be considered as an independent factor in the incidence of this disease.

**Conclusion:**

Aggrecan gene VNTR polymorphism had an association with degeneration of lumbar intervertebral discs that the shorter VNTR repeats increasing the chance of the disc degeneration in this population in the North of Iran. Moreover, an association between the mutant allele (C) of VDR gene *TaqI* polymorphism and disc degeneration is found.

One of the main health issues in men and women between the ages of 20 - 50 years, is low back pain (LBP), which has led to 13 million annual visits in the United States and it costs about 28 million $ per year for the government (1, 2). The intervertebral disk degeneration (IVD degeneration) plays major role in the pathogenesis of the low back pain. Despite the advantages in biomechanics and technology in spinal instruments, minimally invasive surgery and new methods for decompression of spinal cord and roots, the diagnosis and treatment of discogenic back pain is still a major problem (1-3). The concept of degeneration and morphological changes in the intervertebral disc begins with a decrease in notochordal cells before the second decade and it progresses with age. As the aging, remodeling begins in the architecture of the annulus. 

The gelatinous nucleus pulposus becomes dehydrated and fibrotic. Then micro fractures occur at the endplates and in the subchondral surface of vertebral bodies. These changes in annulus and nucleus result in changes in disc morphology, development of clinical symptoms and degeneration appearance in radiologic studies (4-6). Findings of various studies on the risk of degenerative disorder suggest that various environmental and genetic factors can contribute to the development and acceleration of degenerative processes (4, 7, 8). Although environmental factors such as trauma and obesity were previously believed to have contributed, but it seems that genetic factors are more effective than environmental factors in the developing of degenerative phenomena. Some environmental factors like BMI, smoking, trauma and lifting heavy objects, are known as environmental risk factors (8, 9). Different previous studies proposed some genes associated to LBP, among which the most studied candidate genes was vitamin-D receptor (*VDR*) and aggrecan (*ACAN*) as well as interleukin-1 beta (*IL1B*), interleukin-1 alpha (*IL1A*), collagen IX alpha 3 (*COL9A3*) and collagen IX alpha 2 (*COL9A2*). Similar to other complex diseases, it is also difficult to validate the associations between genes with disc degeneration in LBP (9, 10 ).

Although some studies had shown a significant statistic relationship between the VNTR polymorphism of *ACAN *and the *VDR *polymorphism rs731236 (*TaqI*) with intervertebral degeneration, the results were controversial (8, 11-13). Furthermore, to better understand the disease, it would be useful to have information from different populations. So, the aim of this study, was to investigate the relation between aggrecan gene VNTR and *VDR* gene rs731236 (*TaqI*) polymorphisms and lumbar intervertebral disc degeneration in a population in the North of Iran.

## Methods

**The study population:** In this case-control study, 55 patients with symptomatic intervertebral disk degeneration who referred to Sari Imam Khomeini Hospital were selected. Patients had symptoms of low back pain associated with discopathy or radicular pain. Inclusion criteria for the patient group were: patients with discogenic lumbar pain for at least two weeks with or without radicular pain associated with intervertebral disc and having the evidence of degeneration of the intervertebral disc in MRI including, disc dehydration, disc bulging, disc protrusion, disc extrusion signal changes. Control group (n= 55) was selected among the patients with trauma, rather than spinal trauma or under lumbosacral MRI with no evidence of degeneration of one or more intervertebral disc degeneration and patients who did not have any history of current or past dicogenic back pain. Also, the patients admitted to other departments with similar demographic conditions with our patients and had no history of low back pain were selected as the control group. A questionnaire was used to assess medical history, demographic characteristics and personal habits. The results of imaging and severity were evaluated based on the patient's MRI.

An informed consent form was signed by all participants. This project was approved and funded by the Mazandaran University of Medical Sciences (MAZUMS) and also was approved by the ethics committee of the university. 

**Genomic DNA extraction:** A 5 to 10 ml venous blood was obtained from each subject. Lymphocytic genomic DNA was extracted by using “YTA Genomic DNA Extraction mini kit (YektaTajhiz Azma, Iran). The extracted DNA was stored at -20 °C until further processing and study.

**Genotyping of VDR gene polymorphism:** A desired part of *VDR* gene including the rs731236 (*TaqI*) polymorphism was amplified using specific primers (14). A PCR master mix including 11μl distilled water, 0.5μl of each primer at 25μM, 11 μl ready 2x PCR master mix (Amplicon, Denmark) and 2μl template DNA in a total volume of 25μl. The PCR reaction conditions were as follows: 94ºC for 5 minutes, then 35 cycles were applied as 94ºC for 60 seconds; annealing temperature of 60 ºC for 60 seconds,72ºC for 60 seconds. Finally, 1% agarose gel containing SYBR safe staining was used to visualize PCR product. Restriction enzyme *Taq1 *was used to digest the 745 bp PCR product overnight. The digested fragments were 495, 290, 250 and 205 bp in size, which were subjected to electrophoresis on a 1% agarose gel. Wild-type (AA) patients were identified by the presence of 495 and 250 bp fragments. Heterozygous (GA) patients were identified by the presence of 495, 290,250 and 205 bp fragments, while the presence of 290, 250 and 205 bp fragments were the basis for the identification of mutants (GG).

**Genotyping of ACAN gene VNTR polymorphism:** The isolated DNA was used to genotype the variable number tandem repeat (VNTR) of *ACAN* gene via conventional PCR using specific primers (15). Each PCR tube contains 11μl distilled water, 0.5μl of each primer at 25μM, 11 μl ready 2x PCR master mix (Amplicon, Denmark) and 2μl template DNA in a total volume of 25μl. The PCR reaction conditions were as follows: 94ºC for 3 minutes, followed by 35 cycles 94ºC for 45 seconds; annealing temperature of 64 ºC for 45 seconds, 72ºC for 1.30 minutes. A 2% agarose gel containing SYBR safe staining was used to visualize PCR product. The size of alleles was measured according to methods as previously described using a standard molecular weight of 100 bp Plus DNA Ladder (Thermo Fisher Scientific, USA) (15, 16).

**Statistical analysis:** *SPSS* software (Version 23) was used to carry out statistical analysis. The differences of the parametric variables were analyzed by t-test. To compare the nonparametric variables, Mann-Whitney U test was used. Also Hardy-Weinberg equilibrium was tested by chi-square test. Logistic regression was used to calculate odds ratios (ORs) with 95% confidence intervals. Two-tailed p-value less than 0.05 was accepted as statistically significant.

## Results

A total of 110 individuals, including 55 patients with lumbar disc degeneration and 55 controls, were investigated in this study. Epidemiological, demographical and clinical characteristics of the patients and control subjects are shown in table 1. In the patient group, the youngest person was 27 and the oldest was 69 with the mean age of 42.55±1.4 years. In the control group, the youngest person was 18 years old, the oldest was 68 years old and the mean age was 37.35±1.53 years (table 1). Comparing the result between patients and controls with respect to gender, smoking and serum vitamin D levels statistically did not show any significant difference (table 1). Also, the incidence of diabetes mellitus among patients showed significantly higher compared to controls (P=0.03).

**Table 1:** Clinical and demographic data from patients and control subjects

**Variables**	**Case (n=55)**	**Control(n=55)**	**PValue**
Age	42.55±1.4	37.35±1.53	0.013
Sex Male Female	26 (47.3%) 29 (52.7%)	25 (45.5%) 30 (54.5%)	0.84
Smoking	10 (18.2%)	4 (7.3%)	0.15
Diabetes mellitus	13 (24.1%)	5 (9%)	0.03
Serum vitamin D (ng/ml)	26.03±1.70	26.77±1.81	0.75

**Figure 1:**
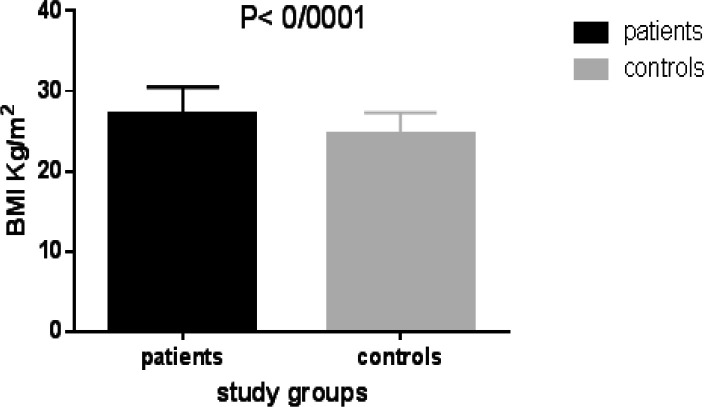
Comparison of BMI between patients and normal controls (n=55)

Body mass index was 27.18 ± 0.45 in case group and 24.64±0.36 in control group respectively. According to Unpaired t-test, the BMI of the case group was higher (figure 1) and the difference between two groups was significant (p=0.001). Also distribution of low back pain in patients group is shown in table 2.

**Table 2:** Distribution of low back pain in patient group (n=55)

**symptoms**	**Cumulative percent**	**Valid percent**	**percent**	**frequency**
Axial back pain	32.7	32.7	32.7	18
Radicular pain	74.5	41.8	41.8	23
Both(axial and radicular pain)	100	25.5	25.5	14

Allele frequency and genotype distributions of vitamin D receptor rs731236 polymorphism are shown in the table 3. The CC genotype (OR=5.337, P= 0.019) was significantly higher among patients, which showed the C allele in patients has a significant higher frequency than in controls (OR= 2.707, P= 0.005). Frequency distribution of VNTR repeats of aggrecan gene in the case and control group is shown in table 5. As can be seen in the table, in the case group, the most frequent alleles were A27, A26 and A24 with a frequency of 52, 12 and 6 numbers in this study, respectively. As shown in table 6, distribution of VNTR repeats in case and control was categorized into two groups as follows: repeats less than or equal to 25 (≤25), and repeats above 25 (>25). The results showed that the group with repetitions ≤25 was related to a 6-fold increase in the incidence of lumbar disc degeneration.

**Table 3:** Genotype and allele frequency of vitamin D receptor rs731236 polymorphism in the case and the control group)n=55)

**Genotype**	**Control (n= 55) (%)**	**Case (n= 55) (%)**	**Odds ratio**	**P-value**
TT (normal)*	42(76.4)	35(63.6)	(95% CI)	
TC	9(16.4)	9(16.4)	1.16 (0.436-3.139)	0.764
CC	2(3.6)	11(20)	5.337(1.327-21.383)	0.019
Allele frequency				
T *	87.80%	71.80%		
C	12.20%	28.20%	2.707 (1.357-5.378)	0.005

* The genotype of TT as well as the allele of T was considered as references.

Frequency distribution of vitamin D receptor gene polymorphism on the basis of smoking, BMI and number of levels involved in MRI is shown in table 4. VAS/NRS (Visual Analogue Scale – Number Pain Rating Scale) scores were performed in the two groups. 

According to the results, the mean pain of patients with repetitions ≤ 25 was 1.94 ± 6 and it was found to be 5.13±1.12 in patients with repetitions over 25, however, the difference was not statistically significant (table 6). Furthermore, distribution of aggrecan VNTR based on BMI, pain severity and smoking are shown in tables 6.

**Table 4:** Frequency distribution of VDR gene polymorphism according to smoking, BMI and number of level involvement in MRI in the patients group

**genotype group**	**TT (Normal)**	**CT (Heterozygous)**	**CC (Mutant)**	**P-** **value **
Non Smoking	29 (64.5%)	7 (15.5%)	9 (20%)	0.94
Smoking	6 (60%)	2 (20%)	2 (20%)	
Normal BMI (18.5-24.9)	10	2	2	
over weight (25 - 29.9)	18	5	4	0.6
obese (≥30 )	7	2	5	
1 level involvement	10	4	4	
2 level involvement	11	4	4	0.58
3 and more levels involvement	14	1	3	

**Table 5:** VNTR repeats of aggrecan gene (ACAN) in case and control group)

**Allele**	**case (n=43)**	**control (n=51)**
18	0	0
19	0	0
20	0	0
21	0	0
22	1	0
23	5	1
24	6	14
25	4	3
26	12	15
27	52	56
28	5	8
29	0	2
30	1	0

**Table 6:** Distribution of aggrecan VNTR in case and control based of BMI, pain severity and smoking

**Variable/ VNTR repeat**	**25≤**	** >25**	**(95% CI) Odds Ratio**	**P- value**
Case	31	8	6.055	0.001
Control	16	25	(2/229 - 16/44)	
pain intensity (mean±SD)	6±1.94	5.13±1.12		0.2
Smoking	6	0		0.31
Non Smoking	25	8		
Normal BMI (18.5-24.9)	6	2		1
over weight (25 - 29.9)	15	4		
obese (≥30 )	10	2		

## Discussion

A number of environmental and demographic factors such as age, sex, smoking, height, and weight were proposed as risk factors for disc degeneration disorders. However, many previous studies have shown the role of different genetic factors and their importance in degenerative processes, including degeneration of intervertebral discs (1, 6, 7).

In this study, the patients were from 27 to 69 years with the mean age of 42.55 with a standard deviation of 1.4. According to previous studies, it is clear that the highest incidence of symptoms and the most frequent referral to doctors for therapeutic or surgical interventions are seen in the third to the fifth decades of the life (17). This study shows that in Iran, the mean age for patients to refer for treatment is similar to the other countries. In a similar study by Eser *et al.* in Turkey on patients with age between 20 and 30 found an association between short repeated alleles of the aggrecan gene VNTR and degenerative disc disease (18). The gender distribution of case group showed 47.30% males and 52.70% females and no significant difference was observed in the incidence regarding gender (table 6). Comparison between smokers/non-smokers in cases and controls showed that 18.2% of the patients and 7.3% controls were smokers. There was no difference between these two groups after statistical analysis. It seems that the findings observed in this study were not affected by gender or smoking (table 6).

The analysis of results by BMI showed that the lowest BMI 21 and 19 and highest BMI were 35 and 32, in case and controls respectively. The descriptive comparison results showed that BMI is more likely to be associated with disk degeneration, and can be considered as an independent risk factor for lumbar disk degeneration. Our results were supported by Cong *et al*. and showed that obesity and VNTR of aggrecan had an association with symptomatic disc degeneration and can be used as a predictor (19). Regarding serum vitamin D levels, 25% (13 individuals) of the patients had a severe deficiency of vitamin D (less than 15mg/dl) and 21% of the controls had severe vitamin D deficiencies, which shows the high prevalence of vitamin D deficiency in the study population (table 6). The pain symptoms were divided into three groups in this study i) radicular pain, ii) low back pain and comorbid low back pain iii) radiculopathy. Based on our findings, radicular pain showed the higher percentage (41.80%) among the patients, but the type of symptoms with the involved disc levels and the age of the patients did not show a significant relationship (table 6).

The number of levels involved in the MRI indicated that a high percentage of patients had one or two levels involved with cumulative percent of 67.30%, however, the highest number of levels involved was 5. In this study, the VAS-NRS was used to assess the severity of pain, which was not considered in previous studies as a risk factor or factor related to the polymorphisms. Accordingly, the relation between the VAS-NRS and the VDR polymorphism was significant. In previous studies, the relation between VDR *TaqI* gene polymorphisms and disc degeneration was investigated. Videman et al. for the first time revealed the association of VDR *TaqI* allele and degeneration of intervertebral disc that is measured by T2-weighted signal intensity (20)[[18. Cheung et al.assessed the effect of the *TaqI* alleles on the risk of developing degenerative disc disease in a Southern Chinese population (5). Previous research revealed that the minor allele (C) of *VDR* *TaqI* is related to a high risk of disc bulge developing and disc degeneration disease, particularly in people less than 40 years. Also, similar studies in Japanese and Finnish population suggest the association of this polymorphism with the development of disc degeneration disease in the lumbar spine (20, 21). However, it should be noted that there are conflicting results regarding the association of this polymorphism with intervertebral disc degeneration. Accordingly, in a meta-analysis, Xu *et al*. demonstrated that the VDR (*TaqI, FokI, ApaI*) polymorphisms were not associated with the risk of this disease(13). Also, the VDR polymorphism was not associated with the number of levels involved in MRI, indicating VD*R* gene *TaqI *allele that did not differ according to the severity of the disease.

Collagen fiber in the central nucleus pulposus of intervertebral disk, is embedded in a highly hydrated aggrecan-containing gel (22).

One of the major proteoglycan of the disk is aggrecan that is responsible for preserving the water content of intervertebral discs and tissue hydration through the osmotic pressure gradient (23, 24). A VNTR polymorphism in exon 12 of the human aggrecan gene encoding the CS1 domain (15) and its most common alleles have 26, 27, or 28 repeats. It is believed that individuals with the longer alleles would be able to produce aggrecan with a greater number of CS chains (25). In this study, the VNTR repeats of *ACAN* gene was between 24 to 28 in controls and 23 to 28 in patients, respectively. Study from different populations revealed a wider range of repeats (18 to 28), however, the most frequent repeats in most studies was 27 (25, 26). Also Solovieva et al. showed that 26 repeats (A26 alleles) were significantly associated with dehydrated discs (27). Different studies by Cong* et al.* demonstrated that repeats less than 25 were associated with symptomatic LDD (19, 26). The study polymorphisms have been introduced in the Asian Caucasians as a risk factor for the disease (28). Results from this study showed that repetitions ≤25 were related to a 6-fold elevation in the chance of degeneration of the lumbar disc. Therefore, comparison of the VDR *TaqI *and the VNTR polymorphism shows that aggrecan gene VNTR polymorphism may be considered as a better predictor than VDR *TaqI *polymorphism in evaluating the risk of IDD. Although, there was no significant difference in pain intensity based on VAS, the mean pain of patient with fewer repeats was more severe than those with more repeats. 

In conclusion** a**ggrecan gene VNTR polymorphism had an association with degeneration of lumbar intervertebral disc, so the shorter VNTR repeats increase the chance of the disc degeneration in this Iranian population. Moreover, there was a significant association between the mutant allele (C) of VDR gene *TaqI* polymorphism and disc degeneration. The study polymorphisms were not associated with the severity of involvement in MRI and pain intensity.
